# Status and Safety Signals of Cephalosporins in Children: A Spontaneous Reporting Database Study

**DOI:** 10.3389/fphar.2021.736618

**Published:** 2021-10-20

**Authors:** Yuanxuan Cai, Linhui Yang, Xiaofang Shangguan, Yuhang Zhao, Rui Huang

**Affiliations:** School of Pharmacy, Tongji Medical College, Huazhong University of Science and Technology, Wuhan, China

**Keywords:** cephalosporin, children, spontaneous reporting system, signal detection, measures of disproportionality

## Abstract

**Introduction:** Cephalosporins are widely used in clinical treatment of children, but it is difficult to carry out clinical trials and there is no strong evidence of their safety. Therefore, adverse drug reactions (ADR) of cephalosporins can be a public health problem that deserves attention.

**Methods:** ADR reports collected by the Hubei Adverse Drug Reaction Monitoring Center from 2014 to 2019 were analysed. The safety of Cephalosporins was described by descriptive analysis and three signal mining methods, including the reporting odd ratio (ROR), proportional reporting ratio (PRR), and comprehensive standard method (MHRA).

**Results:** The findings indicated that the age groups of 0–1 and 2–3 years had the highest rates of reporting ADRs. Children aged 0–4 years reported more ADRs, while the proportion of severe ADRs was lower than the average (6.63%). Among the 37 cephalosporins, the severe ADRs of ceftezole, ceftazidime, cefoperazone/sulbactam, cefotaxime, ceftriaxone were reported more and the proportion of severe ADRs was higher. The proportion of severe ADRs of most cephalosporin compound preparations was higher than that of corresponding single components. A total of 99.18% of the cases improved after treatment. There were four deaths whose ADRs were mainly anaphylactic shock, dyspnoea, and anaphylactoid reaction. In signal mining, the three methods produced 206 signals that were the same, and 73 of them were off-label ADRs.

**Conclusion:** ADRs were common but not serious in children aged 0–4 years. And the reported rate of serious ADRs in children aged over 4 years increased with age. ADR reports of ceftezole, ceftazidime, cefoperazone/sulbactam, cefotaxime, ceftriaxone were numerous and serious, and the safety of cephalosporin compound preparations in children was doubtful. Ceftezole may cause off-label ADRs including tremor, face oedema, cyanosis, pallor, rigors, and palpitation. The labeling of ADRs in children in cephalosporin instructions and the record of allergic history need to be improved.

## Introduction

Cephalosporins belong to β-lactam class of antibiotics and have been developed to the fifth generation at present. Cephalosporins are widely used in the world for their broad antibacterial spectrum, low toxicity, penicillinase resistance and rich varieties. The subsequent adverse drug reactions (ADRs) have also become the focus of public concern. In China, an ADR is defined as the harmful reaction of qualified drugs under normal usage and dosage, which has nothing to do with the purpose of drug use (2011). The annual reports of national ADR monitoring in China from 2017 to 2019 all showed that ADR reports of cephalosporins were the most in the reports of anti-infective drugs, which also had the most reports of serious ADRs (Administration, N.M.P, 2018; Administration, N.M.P, 2019; Administration, N.M.P, 2020).

With the implementation of the two-child policy in 2015 and the three-child policy in 2021, the number of children (0–14) in China has increased rapidly, and the safety of children’s medication has become a key point to improve the health of children. G. M. Park found that antibiotics were the most common ADR causing drugs in children, among which the third cephalosporin was the most common (Park et al., 2012). Jung also believed that antibiotics were the most common drugs that might cause ADRs in children (*n* = 5,159), among which cephalosporins were the most common drugs (*n* = 5,101). Gastrointestinal tract and skin clinical features were the most frequently reported ADR (Jung et al., 2017). In 2015, the National Medical Products Administration warned that cefathiamidine could cause severe ADRs like anaphylactic shock, and a high proportion of ADRs of cefathiamidine have been reported in the ADR reports of children (Administration, S.F.a.D, 2015). Children, as a special medication population, are prone to ADR due to their underdevelopment of liver, kidney and central nervous system and poor ability of metabolism, excretion and tolerance of drugs.

Cephalosporin ADRs in children is a public health problem that deserves attention. It is difficult to carry out clinical trials in children, and there is no strong evidence of safety. And the use of cephalosporins is mostly based on long-term clinical practice. Therefore, it is necessary to re-evaluate the safety of cephalosporins in children after marketing. The purpose of this study was to analyze the provincial spontaneous reporting system (SRS) database to investigate the safety of cephalosporins in children from all aspects of ADRs.

## Materials and Methods

### Data Source and Preprocessing

The data of the ADR reports collected by Adverse Drug Reaction Monitoring Center of Hubei Province from January 2014 to December 2019 were classified and analysed.

The data were cleaned and preprocessed to ensure that they were clean and complete. The ADR database includes all reported ADR reports. Reports of cephalosporin in children aged 0–14 were selected for inclusion. The analysis only included reports with certain, probable, and possible relationships of drugs and ADR evaluated by the reporting unit, and excluded reports that were unlikely or impossible to evaluate. Since there was no unified standard for the entry of drug names and ADRs in the report, the names of active pharmaceutical ingredients (APIs) registered in the National Center For Drug Evaluation were used as the standard to unify the generic names and the ADRs and clinical manifestations were organized according to the World Health Organization Adverse Reaction Terms (WHO-ART).

For the death cases, relevant information was detailed and carefully analysed to find other key points that had contributed.

From January 2014 to December 2019, the ADR Monitoring Center collected a total of 420,114 reports, containing 60,433 reports from children aged 0–14. There were 15,857 reports meeting the inclusion criteria. Since there might be two or more ADRs in a report or case, and the occurrence of an ADR in the use of a certain drug was considered an event, 20,681 events were included in the statistics.

### Data Analysis

A descriptive analysis of sex, age, allergic history, drug, severity, types, and results of ADRs in the reports was carried out.

The amount of each ADR of each cephalosporin was sorted for ADR signal mining, which quantifies the qualitative nature of the relationship between drugs and ADRs (Hauben et al., 2005). In ADR signal mining, the reporting odds ratio (ROR), proportional reporting ratio (PRR), and comprehensive standard method (MHRA) as measures of disproportionality were adopted, which is generally used in this area to detect the imbalance of target events compared with other events in the database (Hauben et al., 2005; Moore et al., 2005). When the frequency of the target drug event combination (DEC) is significantly higher and reaches the threshold compared to the background frequency, a signal is considered to be generated (van Puijenbroek et al., 2002). The strength of the association between drugs and ADRs was expressed as the ROR and PRR with 95% confidence intervals (CIs). The fourfold table used in the measures of disproportionality is shown in Table 1. The calculation formulas and the threshold for generating a signal with these three methods are presented in Table 2. In this study, signal mining of a single drug and a single ADR was conducted without considering the combination of drug use and drug interaction.

**TABLE 1:** The fourfold table used in measures of disproportionality.

Category of drugs	Target ADR N	Other ADRs N	Sum
Target drug	a	b	a+b
Other drugs	c	d	c + d
Sum	a+c	b + d	N = a+b + c + d

**TABLE 2:** Formulas and criteria for generating signals of ROR, PRR, and MHRA.

Method	Formula	Criteria and threshold
ROR	$\text{ROR}=\frac{\left(\text{a}/\text{c}\right)}{\left(\text{b}/\text{d}\right)}=\frac{\text{ad}}{\text{bc}}$	a ≥ 3 and lower limit of 95%CI > 1
	$\text{SE}\left(\ln\text{ROR}\right)=\sqrt{\left(\frac{1}{\text{a}}+\frac{1}{\text{b}}+\frac{1}{\text{c}}+\frac{1}{\text{d}}\right)}$	
	$95\text{\%CI}={\text{e}}^{\ln\left(\text{ROR}\right)\pm 1.96\text{SE}\left(\ln\text{ROR}\right)}$	
PRR	$\text{PRR}=\frac{\text{a}/\left(\text{a}+\text{b}\right)}{\text{c}/\left(\text{c}+\text{d}\right)}$	a ≥ 3 and lower limit of 95%CI > 1
	$\text{SE}\left(\ln\text{PRR}\right)=\sqrt{\left(\frac{1}{\text{a}}-\frac{1}{\text{a}+\text{b}}+\frac{1}{\text{c}}-\frac{1}{\text{c}+\text{d}}\right)}$	
	$95\text{\%CI}={\text{e}}^{\ln\left(\text{ROR}\right)\pm 1.96\text{SE}\left(\ln\text{PRR}\right)}$	
MHRA	$\text{PRR}=\frac{\text{a}/\left(\text{a}+\text{b}\right)}{\text{c}/\left(\text{c}+\text{d}\right)}$	a ≥ 3, PRR ≥ 2, and χ2 ≥ 4
	${\text{χ}}^{2}=\frac{n{\left(\left|ad-bc\right|-\frac{n}{2}\right)}^{2}}{\left(\text{a}+\text{b}\right)\left(\text{a}+\text{c}\right)\left(\text{b}+\text{c}\right)\left(\text{c}+\text{d}\right)}$	

## Results

### Basic Information for ADR Reports

**FIGURE 1:**
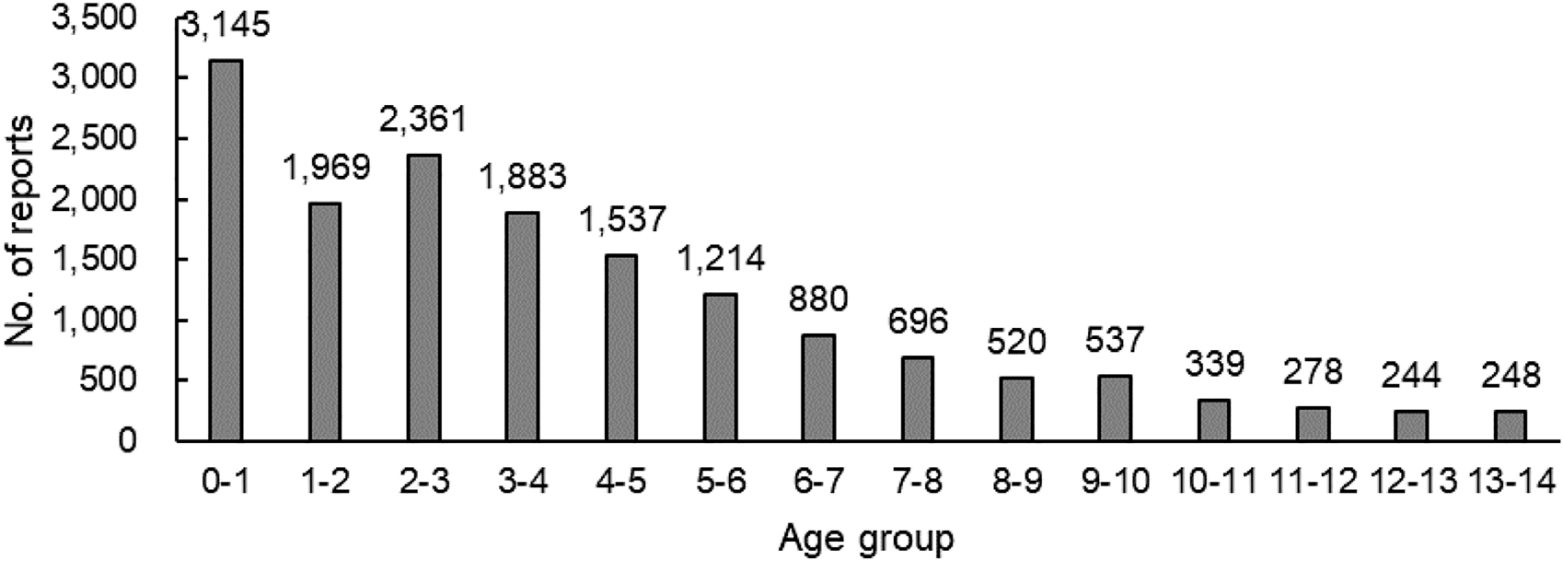
Number of reports in each age group (*n*=15,851).

Among the 15,857 reports related to cephalosporins in children, except for 14 cases in which the sex was unknown, the number of men (9,740) who had ADRs was significantly greater than that of women (6,103), and the male-female ratio was 1.60:1 with a big discrepancy. Excluding six reports of unknown age, the age groups with higher reporting rates were concentrated in 0-1-year-olds (3,145) and 2-3-year-olds (2,361) (see Figure 1). That is, newborns, infants, and young children were the most common.

According to the data that had been recorded, 38 reports were allergic to cephalosporin; 45 reports were allergic to penicillin; 10 reports were allergic to both of them; and 10 reports had a history of alcohol consumption.

### Frequently Reported Cephalosporins

A total of 15,857 cases of cephalosporins in children were reported, which involved 37 kinds of cephalosporins except three cases of unknown cephalosporins. The third cephalosporin was the most reported. Ceftezole, ceftazidime, cefamandole nafate, and cefoperazone/sulbactam were reported in large numbers (see Table 3).

**TABLE 3:** Number of reports of cephalosporins (*n* = 15,857).

Generation	Cephalosporins	N	Generation	Cephalosporins	N
1st	Ceftezole	2,325	3rd	Ceftazidime	1,692
	Cefathiamidine	815		Cefoperazone/Sulbactam	1,565
	Cefazolin pentahydrate	240		Cefotaxime	1,328
	Cefazolin	169		Ceftriaxone	1,128
	Cefazedone	65		Cefoperazone/Tazobactam	924
	Cefadroxil	37		Ceftizoxime	700
	Cefalexin	17		Ceftriaxone/Tazobactam	425
	Cefradine	16		Cefotaxime/Sulbactam	314
	Cefalotin	2		Cefixime	253
	Cefalexin/Trimethoprim	1		Cefmenoxime	97
2nd	Cefamandole nafate	1,589		Cefodizime	93
	Cefuroxime	1,394		Cefoperazone	56
	Cefotiam	231		Cefpiramide	56
	Cefaclor	150		Cefdinir	55
	Cefprozil	23		Cefpodoxime proxetil	6
	Cefuroxime axetil	14		Ceftazidime/Tazobactam	4
	Cefonicid	3		Ceftriaxone/Sulbactam	1
4th	Cefepime	64		Ceftizoxime/Sulbactam	1
	Cefoselis	1	Unknown	Unknown	3

The main route of administration was injection (15,271, 96.30%), followed by oral administration (581, 3.66%).

### Severity of the Reported ADRs

The Administrative Measures on Reporting and Monitoring of ADRs states that according to the severity of ADRs, ADRs were divided into serious and non-serious ADRs. Serious ADRs result in death, life-threatening effects, cancer, a congenital anomaly, birth defects, significant or permanent human disability, damage to organ function, hospitalization or prolonged hospitalization or events that require intervention and treatment to avoid the above results. New and known ADRs are also subdivided according to whether the ADRs are recorded in the drug insert. In addition, ADRs whose types are known but whose severity is greater than that described in the drug insert are also regarded as new ADRs (Administration, 2011) Serious and new ADRs have always been the focus of ADR research, as they pose a greater threat to the life and health of patients.

**FIGURE 2:**
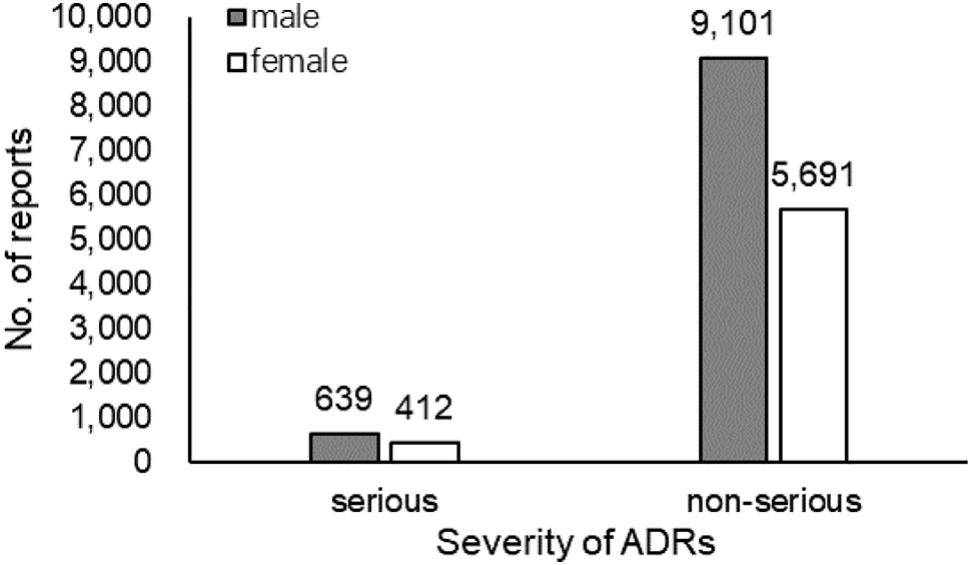
Number of serious and non-serious reports by sex (*n*=15,843).

**FIGURE 3:**
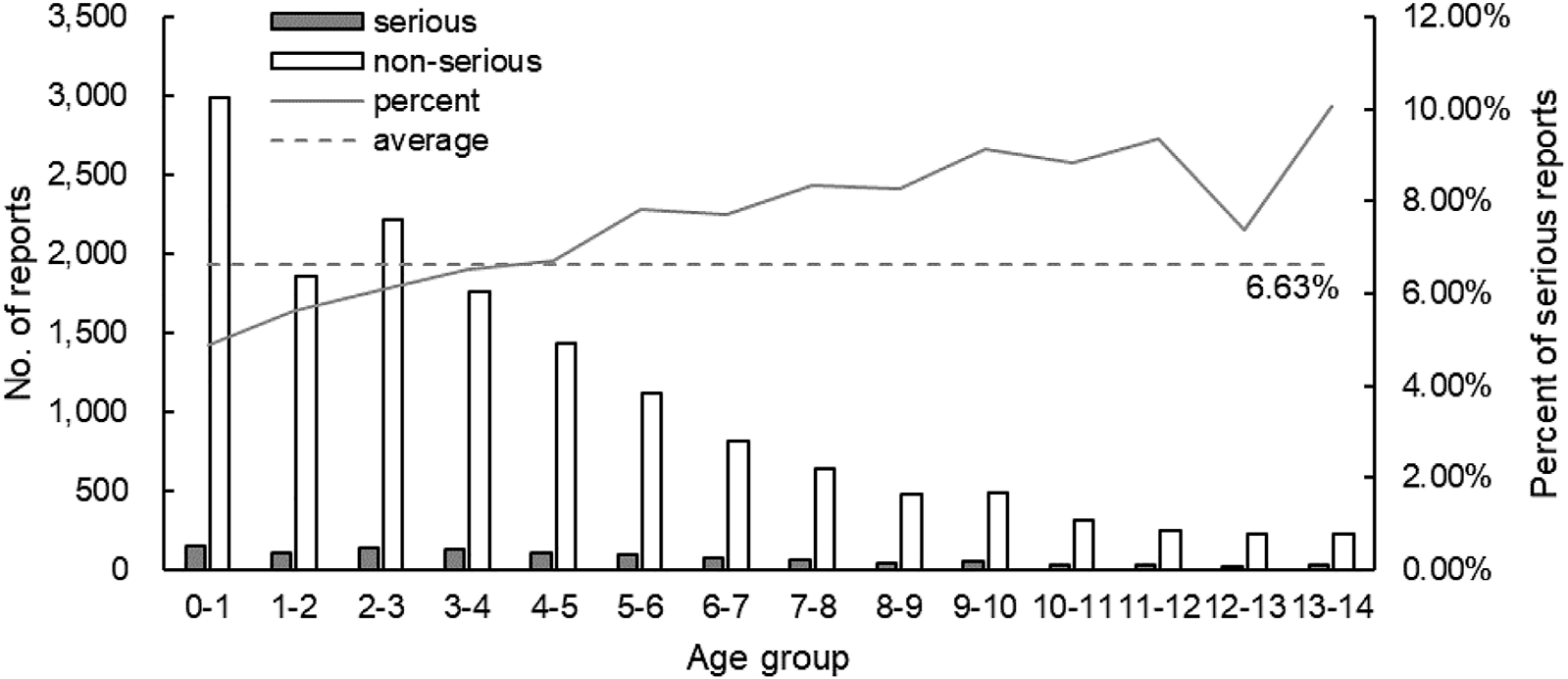
Number of reports and the proportion of serious reports in each age group (*n*=15,851).

Among the 15,857 reports related to cephalosporins in children, there were 1,052 reports of serious ADRs, accounting for 6.63% of the total reports, of which 154 were new and serious reports. There were 14,805 non-serious reports containing 1,573 new and non-serious reports, accounting for 93.37% of the total. The severity of ADRs in males and females was presented in Figure 2. The severity of ADRs was not significantly different by sex (χ2 = 0.219, *p* = 0.640 > 0.05). Figure 3 described serious and non-serious reports in different age groups and the proportion of serious reports, excluding six cases with age unknown. It could be found that the proportion of severe ADRs in children aged 0–4 years was lower than the average (6.63%) although there were more reported ADRs, while the proportion of severe ADRs in children older than 4 years was higher than the average (χ2 = 31.691, *p* = 0.000 < 0.05).

Table 4 shows the number and proportion of serious reports of cephalosporin in children (ordered by the number of reports). Ceftezole, ceftazidime, cefoperazone/sulbactam, cefotaxime, and ceftriaxone had the most severe reports with a higher proportion of severe ADRs. It is worth mentioning that the proportion of severe ADRs of compound preparations was higher than that of corresponding single components except for compound preparations with a small number of reports, such as cefoperazone/sulbactam (9.14% > 7.14%), ceftriaxone/tazobactam (7.76% > 7.27%), and cefotaxime/sulbactam (8.60% > 7.83%).

**TABLE 4:** Number and proportion of serious reports by cephalosporin in children (*n* = 15,857).

Cephalosporins	Serious N (%)	Total	Cephalosporins	Serious N (%)	Total
Ceftezole	168 (7.23)	2,325	Cefazedone	2 (3.08)	65
Ceftazidime	126 (7.45)	1,692	Cefepime	2 (3.13)	64
Cefamandole nafate	73 (4.59)	1,589	Cefoperazone	4 (7.14)	56
Cefoperazone/Sulbactam	143 (9.14)	1,565	Cefpiramide	0 (0.00)	56
Cefuroxime	77 (5.52)	1,394	Cefdinir	2 (3.64)	55
Cefotaxime	104 (7.83)	1,328	Cefadroxil	2 (5.41)	37
Ceftriaxone	82 (7.27)	1,128	Cefprozil	0 (0.00)	23
Cefoperazone/Tazobactam	60 (6.49)	924	Cefalexin	0 (0.00)	17
Cefathiamidine	44 (5.40)	815	Cefradine	1 (6.25)	16
Ceftizoxime	35 (5.00)	700	Cefuroxime axetil	1 (7.14)	14
Ceftriaxone/Tazobactam	33 (7.76)	425	Cefpodoxime proxetil	0 (0.00)	6
Cefotaxime/Sulbactam	27 (8.60)	314	Ceftazidime/Tazobactam	1 (25.00)	4
Cefixime	12 (4.74)	253	Cefonicid	0 (0.00)	3
Cefazolin pentahydrate	13 (5.42)	240	Cefalotin	0 (0.00)	2
Cefotiam	9 (3.90)	231	Cefalexin/Trimethoprim	0 (0.00)	1
Cefazolin	16 (9.47)	169	Ceftriaxone/Sulbactam	0 (0.00)	1
Cefaclor	7 (4.67)	150	Cefoselis	0 (0.00)	1
Cefmenoxime	1 (1.03)	97	Ceftizoxime/Sulbactam	0 (0.00)	1
Cefodizime	6 (6.45)	93	Unknown	1 (33.33)	3

### Frequently Reported ADRs

A total of 20,681 events involved a total of 21 system-organ damage, mainly including skin and appendage disorders, body as a whole-general disorders and gastro-intestinal system disorders. The detailed number and proportion of events were shown in Table 5.

**TABLE 5:** Number and percentage of ADR events related to system-organ damage (top 10, *n* = 20,681).

Rank	System-organ damage	N	Percentage (%)
1	skin and appendages disorders	13,295	64.29
2	body as a whole-general disorders	3,886	18.79
3	gastro-intestinal system disorders	1,703	8.23
4	respiratory system disorders	536	2.59
5	autonomic nervous system disorders	515	2.49
6	central and peripheral nervous system disorders	257	1.24
7	urinary system disorders	151	0.73
8	psychiatric disorders	105	0.51
9	metabolic and nutritional disorders	90	0.44
10	vision disorders	53	0.26

According to the statistics, a total of 153 ADRs were identified, which were concentrated in rash, pruritus, urticaria, maculo-papular rash, allergic reaction, nausea, and vomiting. Table 6 shows the distribution of the number of ADRs in the top 95%. Allergic reaction were the most concerned ADRs, accounting for 15.92%.

**TABLE 6:** Number and proportion of ADRs (*n* = 23,377).

ADR	N	Percentage (%)	Cumulative percentage (%)	ADR	N	Percentage (%)	Cumulative percentage (%)
rash	7,482	36.18	36.18	coughing	182	0.88	89.73
pruritus	3,919	18.95	55.13	erythematous rash	171	0.83	90.56
allergic reaction	3,293	15.92	71.05	face oedema	142	0.69	91.24
urticaria	1,160	5.61	76.66	fever	142	0.69	91.93
vomiting	703	3.40	80.06	rigors	109	0.53	92.46
nausea	390	1.89	81.94	anaphylactoid reaction	108	0.52	92.98
flushing	333	1.61	83.55	cyanosis	108	0.52	93.50
maculo-papular rash	329	1.59	85.15	dizziness	103	0.50	94.00
abdominal pain	287	1.39	86.53	palpitation	101	0.49	94.49
diarrhoea	284	1.37	87.91	dermatitis	101	0.49	94.98
dyspnoea	195	0.94	88.85	agitation	86	0.42	95.39

### Outcome of ADRs

The vast majority of children (99.18%) improved or recovered after treatment and intervention after the occurrence of ADRs. Among the four deaths, two were males and two were females. The children mainly suffered from respiratory and urologic diseases. The main ADRs were anaphylactic shock (2), dyspnoea (1) and anaphylactoid reaction (1) (see Table 7).

**TABLE 7:** Detailed information of the 4 deaths.

case	Sex	Age	Suspected drug	Diseases	Dosage (g)	ADR
1	male	1	Ceftriaxone	upper respiratory tract infection	1	Anaphylactic shock
2	female	5	Ceftriaxone	Urinary tract infection	2	anaphylactoid reaction
3	male	5	Ceftriaxone	acute bronchitis	2	Anaphylactic shock
4	female	14	Ceftazidime	upper respiratory tract infection	2	dyspnoea

### Signal Mining Results

According to the calculation formulas and thresholds, DEC signals that do not meet the criteria were excluded. The ROR generated 211 signals, the PRR generated 207 signals, and the MHRA generated 376 signals. The three signal mining methods produced a total of 206 signals of the same DECs, and some of the signals are shown in Table 8. The larger the ROR and PRR values, the stronger is the correlation between the drug and ADR. All positive signals were sorted into Table 9, and off-label ADRs were marked.

**TABLE 8:** Some of the signals of ADRs (3 methods).

Cephalosporins	ADR	ROR	95% CI lower limit	PRR	95% CI lower limit	χ^2^
Cefadroxil	nausea	51.28	3.2	40.99	3.12	306.65
Cefoperazone/Tazobactam	pneumonia	100.03	3.17	99.64	3.17	147.43
Cefixime	diarrhoea	32.34	3.08	29.28	3.02	710.24
Cefradine	nausea	54.83	3.01	43.13	2.98	164.49
Cefathiamidine	skin disorder	52.41	2.97	52.09	2.97	169.26
Cefazolin	vesicular rash	53.55	2.77	52.75	2.77	92.29
Cefoperazone/Tazobactam	eye pain	89.89	2.71	89.67	2.71	72.25
Cefaclor	diarrhoea	25.58	2.68	23.53	2.64	267.05
Cefprozil	diarrhoea	40.26	2.64	35.19	2.64	99.84
Cefoperazone/Sulbactam	back pain	112.29	2.46	112.12	2.46	56.54
Cefradine	vomiting	30.12	2.41	23.79	2.39	87.73
Cefamandole nafate	abdomen enlarged	106.04	2.4	105.89	2.4	53.29
Cefoperazone/Sulbactam	urticaria acute	18.85	2.37	18.69	2.36	189.1
Ceftriaxone/Tazobactam	eye abnormality	25.44	2.35	25.21	2.35	96.28
Cefalexin	nausea	31.01	2.21	26.91	2.24	51.04
Ceftriaxone	allergic reaction	10.21	2.2	7.54	1.93	2,158.99
Cefuroxime	injection site pruritus	43.63	2.17	43.55	2.17	42.5
Cefoperazone/Sulbactam	anaphylactic shock	14.51	2.16	14.37	2.15	170.13
Cefamandole nafate	rash	9.28	2.14	5.38	1.63	3,394.92
Cefathiamidine	rash	8.97	2.07	5.17	1.58	1,850.75
Cefotaxime	larynx oedema	20.69	2.06	20.62	2.06	63.53
Ceftriaxone/Tazobactam	rash	9.08	2.05	5.14	1.55	1,071.36
Ceftizoxime	diarrhoea	11.31	2.05	10.93	2.02	241.18
Cefuroxime axetil	vomiting	22.79	2.05	19	2.05	51.59
Cefazolin	allergic reaction	10.38	2.04	7.43	1.8	346.89
Cefotaxime/Sulbactam	rash	8.82	1.99	5.05	1.52	767.47
Ceftriaxone	local anaesthesia	18.38	1.98	18.31	1.98	60.13
Cefixime	rash erythematous	14.16	1.97	13.77	1.96	89.4
Cefmenoxime	allergic reaction	10.38	1.95	7.41	1.73	205.47
Cefaclor	dermatitis	19.14	1.94	18.71	1.94	48.55

**TABLE 9:** All positive ADR signals.

Generation	Cephalosporins	Signal N	ADR
1st	Ceftezole	23	tremor^a^, cyanosis^a^, pallor^a^, rigors^a^, palpitation^a^, dizziness^a^, face oedema^a^, agitation^a^, rash, pruritus, erythematous rash, urticaria, urticaria acute, nausea, vomiting, abdominal pain, flushing, sweating increased, fever, hyperpyrexia, coughing, allergic reaction, dyspnoea
	Cefathiamidine	11	dermatitis^a^, coughing^a^, lip disorder^a^, rash, pruritus, urticaria, skin disorder, hyperpyrexia, rigors, oedema, anaphylactoid reaction
	Cefazolin	5	vesicular rash^a^, abdominal pain^a^, nausea, vomiting, allergic reaction
	Cefazolin pentahydrate	5	rash maculo-papular^a^, dermatitis^a^, urticaria^a^, rash, pruritus
	Cefazedone	3	coughing^a^, pruritus^a^, rash
	Cefadroxil	2	nausea, allergic reaction
	Cefradine	2	nausea, vomiting
	Cefalexin	2	nausea, vomiting
2nd	Cefuroxime	16	tremor^a^, injection site pruritus^a^, rash, pruritus, nausea, vomiting, abdominal pain, rigors, fever, hyperpyrexia, dizziness, palpitation, anaphylactoid reaction, allergic reaction, dyspnoea, injection site reaction
	Cefamandole nafate	12	pruritus^a^, coughing^a^, abdomen enlarged^a^, chest pain^a^, face oedema^a^, agitation^a^, rash, urticaria, skin disorder, fever, hyperpyrexia, injection site reaction
	Cefaclor	7	dermatitis^a^, abdominal pain^a^, flushing^a^, vomiting, nausea, diarrhoea, allergic reaction
	Cefotiam	6	face oedema^a^, rash, rash erythematous, urticaria, flushing, fever
	Cefprozil	3	nausea, vomiting, diarrhoea
	Cefuroxime axetil	1	vomiting
3rd	Cefoperazone/Sulbactam	17	flushing^a^, dyspnoea^a^, agitation^a^, dermatitis^a^, dizziness^a^, palpitation^a^, back pain^a^, cyanosis^a^, rash, pruritus, rash maculo-papular, urticaria acute, nausea, vomiting, abdominal pain, allergic reaction, anaphylactic shock
	Ceftazidime	15	pallor^a^, flushing^a^, cyanosis^a^, rigors^a^, increased stool frequency^a^, rash, pruritus, rash maculo-papular, rash erythematous, urticaria, urticaria acute, nausea, vomiting, abdominal pain, allergic reaction
	Cefotaxime	14	rash maculo-papular^a^, dermatitis^a^, vesicular rash^a^, palpitation^a^, larynx oedema^a^, lip disorder^a^, rash, pruritus, nausea, vomiting, headache, allergic reaction, anaphylactic shock, dyspnoea
	Cefoperazone/Tazobactam	10	vesicular rash^a^, coughing^a^, pneumonia^a^, face oedema^a^, eye pain^a^, eye abnormality^a^, agitation^a^, rash, pruritus, urticaria
	Ceftriaxone	9	dyspnoea^a^, anaesthesia local^a^, pain^a^, pruritus, rash erythematous, abdominal pain, anaphylactoid reaction, allergic reaction, anaphylactic shock
	Ceftizoxime	9	rash maculo-papular^a^, flushing^a^, pallor^a^, rigors^a^, cyanosis^a^, oedema periorbital^a^, rash, diarrhoea, allergic reaction
	Cefixime	7	rash, rash erythematous, urticaria, nausea, diarrhoea, flushing, sweating increased
	Cefotaxime/Sulbactam	6	anaphylactoid reaction^a^, face oedema^a^, oedema^a^, eye abnormality^a^, rash, pruritus
	Ceftriaxone/Tazobactam	5	coughing^a^, eye abnormality^a^, rash, pruritus, urticaria
	Cefodizime	4	rash, pruritus, rash maculo-papular, allergic reaction
	Cefmenoxime	3	rash maculo-papular^a^, rash erythematous, allergic reaction
	Cefoperazone	3	rash, rash erythematous^a^, diarrhoea
	Cefpiramide	2	rash, allergic reaction
	Cefdinir	2	rash, diarrhoea
4th	Cefepime	2	diarrhoea, allergic reaction
Total		206	—

a Off-label ADRs.

## Discussion

According to statistics, in recent 6 years, the ADR reports of cephalosporins in children aged 0–14 reported by SRS were mainly concentrated in children aged 0–3. Jiang found that among the people who had ADRs after taking cephalosporins, young children were the most prominent (Jiang et al., 2021). Zheng’s investigation on a hospital found that the number of children with ADRs to cephalosporins was mainly 0–3 years old (Zheng et al., 2018). The above results were consistent with the results of this study, which suggested that the physiological function of children, especially newborns and infants, was not fully mature; drug metabolism was slow; and drug accumulation was easy to occur, resulting in a high incidence of ADR. Due to the limitations of data collection, it was not possible to know the frequency of cephalosporin use by age group.

The patient’s allergy history may be incomplete, making it difficult to make a meaningful analysis. However, a study involving 13,153 cases of cefazolin skin testing in South Korea found that 15% of patients with a history of β-lactam allergy were positive for the skin test; 1.35% of the patients without a history of β-lactam antibiotic allergy were positive, indicating that the history of β-lactam allergy may be associated with the occurrence of cephalosporin allergic reactions (Kwon et al., 2019). In addition, there may also be cross-reactivity in cephalosporin allergy (Li et al., 2019). Perfecting the records of allergic history will be helpful to the prediction of allergic reactions.

Combined with the severity of ADRs, the results showed that there was no significant difference in the distribution of the severity of ADRs between different sexes. Notably, although there were many ADR reports in children aged 0–4 years, the proportion of severe ADRs is the lowest. The likely reason was that doctors were more cautious in using cephalosporins when treating newborns and infants, prioritizing safety over efficacy (Lu et al., 2018). The reported rate of severe ADRs generally increased with age. Children at this age were in a period of rapid growth and development, and their physical conditions fluctuated greatly, so it was difficult to determine the appropriate dose. This may have something to do with the difficulty in accurately estimating the appropriate dose from experience and the increasing confidence of doctors in medication as children age. It was suggested that doctors strictly followed the drug instructions and antibiotic medication guidelines, comprehensively analyzed the state of the children, strictly controlled the dosage and prevented the inducing of drug resistance.

This study found that the use of cephalosporin compound preparations in children was more prominent in severe ADRs, and the proportion of severe ADRs in most cephalosporin compound preparations was higher than the average. At present, there were few studies on ADRs of cephalosporin compound preparations, among which the research of cefoperazone/sulbactam was the most abundant. In China, a number of retrospective studies on efficacy and ADRs reported that the efficacy of cefoperazone/sulbactam was significantly higher than that of ceftazidime in the control group, and the incidence of ADRs was considered lower than that of the control group (Zhu, 2019; Wang et al., 2020). The subjects in these studies were all older than 20 years. The noninferiority trial conducted by Liu on pneumonia patients over 18 years old showed no significant difference in the mortality rate and proportion of severe ADRs in the cefoperazone/sulbactam group compared with the cefepime group, suggesting that the two groups had the same efficacy and safety (Liu et al., 2019). In adults, ADRs and severe ADRs caused by cephalosporin compound preparations appeared to be no different or better than those caused by other conventional cephalosporins. Few studies have been conducted on ADRs of cephalosporin compound preparations in children. But the only studies that have been done on children seem to come to a different conclusion than adults, Pareek et al. compared the efficacy and safety of cefotaxime/sulbactam with amoxicillin clavulanate (conventional treatment) and found that one patient in the cefotaxime/sulbactam group reported a severe ADR to convulsion, except that both drugs were safe and well tolerated in the study population (Pareek et al., 2008). A clinical study involving 986 patients treated with cefotaxime/sulbactam found a higher incidence of ADRs in children than adults (12.73 vs. 6.46%, *p* < 0.05) (Chen et al., 2017). The ADRs of cephalosporin compound preparations in children may have different characteristics from that of adults. In addition, the pharmacokinetic trials of cephalosporin compound preparations in human volunteers were conducted in healthy adults, and the results showed no pharmacokinetic interaction between the two components, but it was not clear whether the it was consistent in children (Ma et al., 2003; Mingjie et al., 2008; Yang et al., 2011). ADRs, severe ADRs, factors and other aspects related to the safety of cephalosporin compound preparations in children may need more and in-depth studies.

In this study, it was found that the proportion of severe ADRs of most cephalosporin compound preparations was higher than that of the corresponding single formulations. Cephalosporins in compound preparation can effectively prevent bacteria from synthesizing cell wall and inhibit bacterial division, but is easily hydrolyzed by β-lactamase. Sulbactam and tazobactam are β-lactamase inhibitors, which can inhibit the activity of hydrolase but have weak antibacterial effect. Combined use of the two can increase the stability of cephalosporins and enhance the antibacterial effect. Many *in vitro* and *in vivo* experiments at earlier times have confirmed that cephalosporin compound preparations have better antibacterial effect than the corresponding single preparations (Crosby and Gump, 1982; Knapp et al., 1990; Fu et al., 2002; You et al., 2003; Prakash et al., 2005; Yong et al., 2006; Li et al., 2010). However, no comparative studies on safety between compound preparations and single preparations have been found, and only comparative studies on toxicity reactions were identified. Li investigated the difference of toxicity reaction between cefoperazone/tazobactam and single component. Acute toxicity test showed no abnormal reaction and no death in the tested animals; The long-term toxicity test showed no significant differences in hematology, blood biochemistry, coefficient of vital organs and pathology between the cefoperazone/tazobactam group and the single component group (Li et al., 2003). This study suggested that the use of cephalosporin compound preparations in children may increase the efficacy as well as ADR compared with the single preparations, probably because the impurity profile of compound preparations is not the simple summation of impurity profile of single formulations, but there are more new impurities and change quickly, indicating more allergic reactions.

ADRs of Cephalosporins in children mainly involved skin and accessory damage, systemic damage and gastrointestinal system damage, including rash, pruritus, urticaria, allergic reaction, vomiting, and nausea. There were only a few ADRs such as liver function damage, hematuria, and leucopenia. Misreporting was a common problem in SRS. Reactions of skin, gastrointestinal tract and the whole body were easy to detect, while ADRs related to liver, kidney, and blood may need to be reflected by biochemical indicators. And it was possible that medical institutions and doctors may choose not to report serious ADRs out of self-interest.

After treatment and intervention, ADRs of most patients have been improved or cured, but a few patients still left sequelae or died. Anaphylactoid reaction and anaphylactic shock accounted for the majority of the death reports, among which three cases of death reports used ceftriaxone from the same manufacturer. So it cannot be ruled out that ADRs may be caused by product quality problems. Besides, the safety of ceftriaxone has been a prominent problem. In the pharmacovigilance database of Iran from 1998 to 2009, ceftriaxone had the highest number of deaths (49 cases) (Shalviri et al., 2012); Ceftriaxone was the main drug in 112 cases of anaphylactic shock reported by SRS of Republic of Crimea from 2010 to 2018. Some literature analysis on severe ADRs of ceftriaxone showed that there were more cases of severe allergic reaction and anaphylactic shock, and anaphylactic shock was the main cause of death (Zhang et al., 2010; Lu et al., 2011). The safety of ceftriaxone still needs extra attention. Doctors should strictly follow the medication indications, strengthen the monitoring and treatment of allergic reactions, and actively carry out anti-allergy treatment.

In this study, ADR signals obtained from signal mining in the background of medication use in children may have child specificity. Combined with the drug instructions, 73 off-label ADRs were found. The off-label ADR signals of ceftezole with the largest number of reported cases were analyzed one by one.

De-Sarro reported that ceftezole was characterized by the presence of a tetrazole nucleus similar to pententytetrazole at position seven, and thus has convulsion activity. Tremor, convulsion and limb spasm occurred in both rats and dogs after intravenous administration (De Sarro et al., 1995). No cases of tremor after using ceftezole have been found in children, but a documented case of tremor and convulsion after intravenous ceftezole in an adult woman with uremia has been reported (Jin, 2009). Due to uremia, drug excretion was slowed down; plasma half-life was prolonged; the blood-brain barrier was damaged; and drugs accumulated in the central nervous system. Patients with uremia were more likely to develop antibiotic encephalopathy, or in severe cases of grand mal epilepsy. Given the six cases reported in this study and the fact that renal function and blood-brain barrier were not fully developed in children, tremor may be associated with the drug.

The occurrence of dizziness appeared to be a rare ADR of ceftezole, and few cases have been identified in related studies. When Geng evaluated the efficacy of a drug in children with ceftezole as the control group, dizziness occurred in three out of 40 cases (Geng, 2017). In the drug efficacy studies without age limits, Wang found dizziness in four cases (*n* = 40) and Yang found dizziness in three cases (*n* = 136) (Yang et al., 2010; Wang and Zeng, 2021). However, the specific mechanism of ceftezole induced dizziness remained unclear and more research was needed.

No facial edema has been reported with ceftezole. In this study, facial edema was mainly presented as facial and eyelid edema. Given the positive signals and description of the reported data, more studies were needed.

Anxiety with ceftezole was not common. Ma used the SF-36 score to evaluate the mental state of patients (without age limit) after the use of ceftezole. The higher the score, the better the state. The study obtained a low mental state score of 61.29 (Ma, 2018). It was difficult to judge whether the anxiety and restlessness was caused by the drug, because it seemed understandable that the child was anxious in an unfamiliar environment and in a state of physical discomfort.

Reports of ADRs such as cyanosis, pallor, chill, and palpitation with ceftezole have occasionally been seen. Wei analyzed 113 cases of ADRs of ceftezole in a hospital, and found two cases of chill (Jiao et al., 2010). Guo reported a case of elderly patients with sudden palpitations, pallor, and cold extremities after the injection of ceftezole (Guo and Sun, 2005). Since there were few studies related to children, there were no reported cases of these ADRs in children, and the relevant mechanism studies were even less.

In cephalosporin instructions, ADRs are well documented, most of which include the data of clinical trial and passive monitoring. In the process of examining the instructions, it was found that the ADR items of each cephalosporin were approximately consistent with the statistics for the number of ADRs in the study. However, there are few ADR annotations and clinical trial reports of ADRs related to medication in children in cephalosporin instructions. Due to economic and ethical issues, clinical trials of medicines for children are limited, resulting in a lack of efficacy and safety data for children. Some European countries have introduced relevant policies and regulations that allow manufacturers to enrich clinical trials of drugs with children as research subjects when conditions permit (Marinovic et al., 2016; Ciato et al., 2017). In addition, ADR signals of cephalosporins with children’s particularity could be obtained by data mining, which could be used as data support of ADRs in the instructions to enrich the label of ADRs related to medication in children.

The statistical results and ADR signals obtained in this study are helpful in guiding the safe use of cephalosporins for children in the clinic, and might be clues for ADR mechanism research, even providing advice for modifying drug labels based on results that may be special to children and the detection of off-label ADRs. In addition, this study has potential limitations. The effect estimated in the study is based on the data of a single province. Although the data are considerable, the external validity of the conclusion still needs to be improved. Due to the limitation of the selected signal mining method, the combination of drugs is not considered, and the conclusions may be biased.

## Conclusion

ADRs were common but not serious in children aged 0–4 years. And the reported rate of serious ADRs in children aged over 4 years increased with age, possibly because the body fluctuated greatly at this stage and it was difficult to determine the appropriate dose with empirical medication. ADR reports of ceftezole, ceftazidime, cefoperazone/sulbactam, cefotaxime, ceftriaxone were numerous, and serious, which deserved attention. Studies on the safety of cephalosporin compound preparations in children were few, and the safety of cephalosporin compound preparations in children was doubtful. ADR signal mining was helpful to identify off-label ADRs. Ceftezole may cause off-label ADRs including tremor, face oedema, cyanosis, pallor, rigors, and palpitation. It was also found that the labeling of ADRs in children in cephalosporin instructions and the record of allergic history need to be improved.

## Data Availability

The data analyzed in this study is subject to the following licenses/restrictions: The data is provided by Adverse Drug Reaction Monitoring Center of Hubei Province and the data is not publicly available due to institutional confidentiality requirements. Requests to access these datasets should be directed to RH, hys19810612@163.com.
